# Sleep quality assessment in chronic rhinosinusitis patients submitted to endoscopic sinus surgery: a meta-analysis^☆^

**DOI:** 10.1016/j.bjorl.2019.06.008

**Published:** 2019-07-29

**Authors:** Manuela Dowsley A. Guttemberg, Fabiana A. Figueiredo da Mata, Márcio Nakanishi, Keitty R.C. de Andrade, Maurício G. Pereira

**Affiliations:** a Universidade de Brasília (UnB), Faculdade de Medicina, Brasília, DF, Brazil; b Hospital Universitário de Brasília, Divisão de Cirurgia, Departamento de Otorrinolaringologia – Cirurgia de Cabeça e Pescoço, Brasília, DF, Brazil

**Keywords:** Chronic rhinosinusitis, Sleep quality, Endoscopic sinus surgery, Meta- analysis, SNOT-22, Rinossinusite crônica, Qualidade do sono, Cirurgia endoscópica nasossinusal, Meta-análise, SNOT-22

## Abstract

**Introduction:**

Chronic rhinosinusitis can lead to poor sleep quality in affected individuals. Endoscopic nasal surgery has been indicated for patients with chronic rhinosinusitis, resulting in improved quality of life, but it is still unknown if there is a similar improvement in sleep quality after the surgical procedure.

**Objective:**

To estimate the sleep quality of patients with chronic rhinosinusitis after undergoing endoscopic sinus surgery.

**Methods:**

The literature search was conducted in the indexed databases PubMed, Embase, Lilacs, SciELO, Google Scholar, Web of Science, Scopus, Database of Thesis and Dissertations of CAPES, Cochrane Library, Clinical Trials and in the grey literature. It included studies that reported the sleep quality of patients with chronic rhinosinusitis after undergoing endoscopic sinus surgery based on questionnaires assessing quality of life. Two researchers independently conducted the study selection and extraction. The random effects model was chosen to conduct the meta-analysis that was performed using the statistical package STATA, version 11.

**Results:**

Overall, 4 studies and 509 subjects were included in the systematic review. Improved sleep quality was observed in 90% of the patients. There was an improvement (on average, from 57% to 67%) in each of the five symptoms related to sleep quality. The results of the meta-analysis revealed high heterogeneity.

**Conclusions:**

This review shows that a large percentage of patients report improved sleep quality after endoscopic sinus surgery.

## Resumo

**Introdução:**

A rinossinusite crônica pode levar a uma má qualidade do sono nos indivíduos afetados. A cirurgia endoscópica nasal tem sido indicada para pacientes com rinossinusite crônica, resulta em melhoria da qualidade de vida, mas ainda não se sabe se há melhoria semelhante na qualidade do sono após o procedimento cirúrgico.

**Objetivo:**

Estimar a qualidade do sono em pacientes com rinossinusite crônica após serem submetidos à cirurgia endoscópica nasossinusal.

**Método:**

A busca na literatura foi feita nas bases de dados indexadas PubMed, Embase, Lilacs, SciELO, Google Scholar, Web of Science, Scopus, Banco de Teses e Dissertações da Capes, Cochrane Library, Clinical Trials e na literatura cinzenta. Foram incluídos estudos que relataram a qualidade do sono de pacientes com rinossinusite crônica após ser submetidos à cirurgia endoscópica nasossinusal, com base em questionários que avaliaram a qualidade de vida. Dois pesquisadores conduziram independentemente a seleção e extração dos estudos. O modelo de efeitos aleatórios foi escolhido para conduzir a meta-análise que foi feita com o pacote estatístico STATA, versão 11.

**Resultados:**

No total, 4 estudos e 509 indivíduos foram incluídos na revisão sistemática. Melhora na qualidade do sono foi observada em 90% dos pacientes. Houve melhora (em média, de 57% a 67%) em cada um dos cinco sintomas relacionados à qualidade do sono. Os resultados da meta-análise apresentaram alta heterogeneidade.

**Conclusões:**

Esta revisão mostra que uma grande porcentagem de indivíduos relata melhoria na qualidade do sono após a cirurgia endoscópica nasossinusal.

## Introduction

Chronic rhinosinusitis is one of the most prevalent chronic diseases in the United States of America (USA) and in Europe. It is believed that the disease affects 31 million people per year in the United States,^1^ affecting approximately 15% of the adult population.^2^ In the Global Allergy and Asthma European Network project (GA2LEN), the prevalence rate of chronic rhinosinusitis was found to reach 10.9% in the European population.^3^

It is estimated that poor sleep quality affects approximately 70 million Americans each year.^4^ Moreover, patients with chronic diseases such as chronic rhinosinusitis have a higher prevalence of sleep dysfunctions than that observed in the general population.^5^ Sleep disturbances lead to changes in the quality of life and high public health care costs and indirect costs, such as decreased work productivity and absenteeism.^6^

Since the nose is the first port of entry for inspired air under normal conditions, nasal pathologies have a significant impact on airflow and potentially contribute to sleep-related respiratory disorders.^7^, ^8^ Recently, it has been shown that over 75% of the patients with chronic rhinosinusitis report abnormal sleep quality, with worse sleep in those with more severe sinus disease.^9^

Endoscopic sinus surgery has been indicated for patients with chronic rhinosinusitis failing improvement by clinical drug treatment. In these cases, the improvement in the Quality of Life (QL) after surgery is evident.^10^, ^11^ However, whether there is a similar improvement in sleep quality after endoscopic sinus surgery is still unknown.^12^

Tools for assessing the quality of life and sleep are available in the literature.

For example, the Sino-Nasal Outcome Test 22 (SNOT-22) is an update of the Sino-Nasal Outcome Test-20 (SNOT-20) questionnaire^13^ and is a specific tool for evaluating the QL in sinonasal diseases. It is also used to compare the quality of life in the pre- and postoperative periods of endoscopic sinus surgery in patients with chronic rhinosinusitis. In these questionnaires, five of the questions are directly associated with sleep quality.

Given the relevance of chronic rhinosinusitis as a public health problem and its association with sleep diseases, this study aims to investigate the improvement in sleep quality after surgical treatment for chronic rhinosinusitis through a systematic review and meta-analysis.

## Methods

The Preferred Reporting Items for Systematic Reviews and Meta-Analysis (PRISMA) statement and checklist were used during this review.^14^

### Registered protocol

The review protocol was registered in the International Prospective Register of Systematic Reviews (PROSPERO) database under registration number CDR 42016036536.

### Eligibility criteria

To be eligible for this systematic review, studies had to be conducted with adults (18 years of age and over) diagnosed with chronic rhinosinusitis and had to provide the average scores of sleep quality on life and sleep questionnaires before and after the surgical procedure.

Studies that did not provide the necessary data to calculate the percentage of people reporting a sleep quality change after surgery were excluded. Studies were also excluded when the sleep quality scores were part of the overall quality of life scores and it was not possible to extract only the scores related to sleep quality.

### Data sources and search strategy

The literature search was conducted between the 01^st^ and 04^th^ of September 2016 in the following electronic databases: Medline (via PubMed), Embase, Lilacs, SciELO, Google Scholar, Web of Science, Scopus, Cochrane Library, Clinical Trials and CAPES (the Brazilian Ministry of Education database). There were no restrictions concerning language, date or publication status.

The search strategy employed Medical Subject Headings (Mesh) terms in PubMed, EMTREE terms in Embase and a group of keywords. As an example, the following search strategy as used for searching in MEDLINE (via PubMed): (“sinusitis” [mesh] OR “sinusitis” [tiab] OR “chronic rhinosinusitis” [tiab] OR “rhinosinusitis” [tiab]) AND (“sleep” [mesh] OR “sleep” [tiab] OR “sleep quality” [tiab] OR “sleep disorder” [tiab] OR “poor sleep” [tiab] OR “apnea” [tiab] OR “sleep disturbances” [tiab] OR “nighttime awakenings” [tiab] OR “PSQI” [tiab] OR “quality of sleep” [tiab] OR “SNOT-22” [tiab] OR “berlim” [tiab] OR “EpSS” [tiab] OR “Epworth” [tiab] OR “RSDI” [tiab]) AND (“ESS” [tiab] OR “endoscopic sinus surgery” OR “Surgical Treatment” [tiab]).

The search strategy was adapted to the specific criteria for each database.

### Study selection

Two researchers independently managed the study selection and eventual disagreements were resolved by consensus. Initially, articles were pre-selected based on their titles and abstracts. Duplicate articles were excluded. The complete texts of the pre-selected records were read independently by the two researchers, and the articles that met the eligibility criteria were included for review.

### Quality assessment

The studies’ quality assessment was performed based on the tool “Quality Assessment Tool for Before-After (Pre-Post) Studies With No Control Group” from the NIH (National Heart, Lung and Blood Institute).^15^ This instrument includes twelve items for critically evaluating the methodological quality of articles that report data in before-and-after studies. For each obeyed item, the study received a “YES”. The higher the total number of “YES” answers for a study was, the lower the risk of bias attributed to it was.

### Data collection process

Two reviewers independently extracted the data from the selected studies to a standard Microsoft Excel 2010 worksheet. In cases of disagreement, the decision was made by consensus.

Extracted data included author names, year of publication, research period, place of study, study design, sample size, average age, sleep improvement after the surgical procedure and follow-up time of the participating patients. Authors of the selected articles were contacted in an attempt to collect additional information to insure that the data sheet was completed as thoroughly as possible.

In cases that more than one instrument measuring sleep quality was used (Epworth Sleepiness Scaler or Pittsburgh Quality Index), only data for SNOT-20 and SNOT-22 were extracted for review. In addition, the small number of articles found that included the others sleep quality questionnaires (less than five) would compromise the performance of the meta-analysis calculations. This strategy was used in an attempt to preserve homogeneity in the studies.

Data on sleep quality before and after surgery were extracted from the SNOT-20 and SNOT-22 questionnaires. Both questionnaires measure quality on a scale from 0 to 5 in the five specific questions of the sleep domain: difficulty falling asleep, wake up at night, lack of a good night´s sleep, wake up tired and fatigue. Zero indicates no problem, and five indicates a problem as bad as possible. We considered improvement to be at least a 1 point decrease on the scale.^16^

### Data management and statistical analyses

The main outcome of this review was the percentage of individuals with chronic rhinosinusitis who reported improved sleep quality after undergoing endoscopic sinus surgery.

### Meta-analysis

The random effects model was chosen to conduct the meta-analysis. In the analysis, the “metaprop ftt*”* command of the STATA statistical package was used because it incorporates the Freeman-Tukey double arcsine transformation capable of stabilizing the variances between the studies.^17^, ^18^ The chi-square test (*p* < 0.10) was calculated to test the heterogeneity between studies. The Chi-Square method is considered a low power test when few studies or studies of small samples are taken for analysis; therefore, to be more conservative, *p* < 0.10 was chosen instead of the standard *p* <  0.05.^19^

### Sleep quality scores before and after the surgery

The average scores in each of the five items concerning sleep quality were compared before and after endoscopic sinus surgery to calculate the percentage of improvement in each of the items. In the studies that provided enough data, it was also possible to analyse the score averages in the sleep domain as a whole (summing of the five items related to sleep before and after the surgery).

## Results

### Selection process and study characteristics

**Fig. 1:**
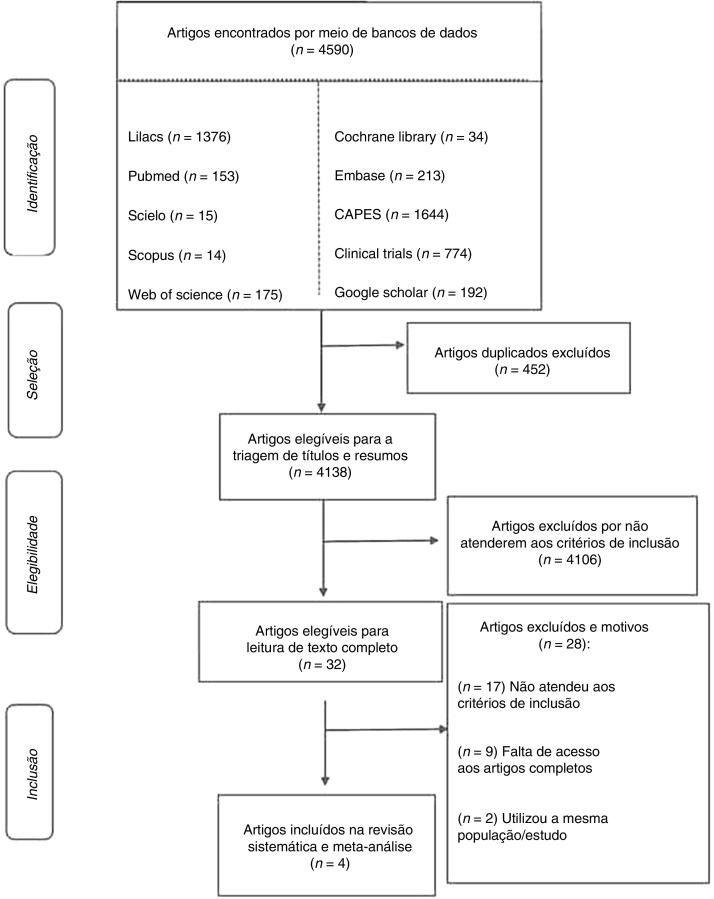
Flow diagram of the study selection process.

The search in the databases resulted in 4,590 registers and 32 studies being selected for full-text review after the removal of duplicate studies and evaluation of the titles, abstracts and inclusion criteria. Fig. 1 details the selection process and the reasons why the registers were excluded. Overall, 4 studies and 509 patients were included in the review (Table 1).^12^, ^20^, ^21^, ^22^

**Table 1:** Study characteristics.

Author, year of publication	Alt et al.^12^	De Vilhena et al.^21^	Mascarenhas et al.^22^	Li et al.^20^
Local	Oregon Helth & Sciece University (Portland, OR) Medical University of South Carolina (Charleston, SC) Stanford University (Palo Alto, CA) University of Calgary (Alberta, CA)	Hospital Pedro Hispano (Porto, Portugal).	UNIFESP-EPM (São Paulo, Brasil).	Central South University, Changsa, China.
Period of data collection	April 2011 to January 2014	September 2012 to February 2014	NR	April to October 2011
Sample characteristics	Adult patients (18 years and over) with a diagnosis of refractory CRS. Exclusion: Acute Repetitive Rhinosinusitis, sleep apnea or corticoid dependence.	Adult patients with CRS with polyps. Exclusion: Previous nasal surgery or not complete questionnaire.	Adult patients (18 years and over) with a diagnosis of CRS with or without polyps.	Adult patient’s diagnosis of refractory CRS with or without polyps. Exclusion: Patients with asthma and ASA intolerance.
Type of study	Before and After	Before and After	Before and After	Before and After
Sample size (n)	219	100	38	152
Age, mean	50.7 (±14.7)	42.8 (±14.9)	46.2	35.2 (±12.3)
Female (%)	118 (53.9%)	45 (45%)	22 (57.9%)	64 (42.1%)
Sleep improvement in the sample (%)	72%	99%	92.1%	NR
Follow-up time (months)	6	3	3 e 24	3, 6 e 12
Diagnosis of CRS	2007 Adult Sinusitis Guideline (AAO-HNS)	NR	EPOS 2012	NR
Quality of Life questionnaire	SNOT-22	SNOT-22	SNOT-22	SNOT-20

CRS, Chronic Rhinosinusitis; EPOS 2012, European Position Paper on Rhinosinusitis and Nasal Polyps 2012; NR, Not Reported; SNOT-20, Sino-Nasal Outcome Test-20; SNOT-22, Sino-Nasal Outcome Test-22; UNIFESP, Universidade Federal de São Paulo.

Three studies used the SNOT-22, and only one used the SNOT-20. One study was conducted in North America (Canada and the United States), one in South America (Brazil), one in Europe (Portugal) and one in Asia (China).

All articles were evaluated as having good/moderate quality, with the approximate number of 7 “YES” answers from 12 answers in total per study (Table 2).^12^, ^20^, ^21^, ^22^ The four studies were included in the meta-analysis of this review.

**Table 2:** Quality assessment of the studies.

Quality questions	Author, year of publication			
	Alt et al.^12^	De Vilhena et al.^21^	Mascarenhas et al.^22^	Li et al.^20^
1. Was the study question or objective clearly stated?	YES	YES	YES	YES
2. Were eligibility/selection criteria for the study population prespecified and clearly described?	YES	YES	YES	NO
3. Were the participants in the study representative of those who would be eligible for the test/service/intervention in the general or clinical population of interest?	CD	CD	CD	CD
4. Were all eligible participants that met the prespecified entry criteria enrolled?	YES	YES	YES	CED
5. Was the sample size sufficiently large to provide confidence in the findings?	YES	CD	CD	CD
6. Was the test/service/intervention clearly described and delivered consistently across the study population?	YES	NO	NO	YES
7. Were the outcome measures prespecified, clearly defined, valid, reliable, and assessed consistently across all study participants?	YES	YES	YES	YES
8. Were the people assessing the outcomes blinded to the participants' exposures/ interventions?	NR	NR	NR	YES
9. Was the loss to follow-up after baseline 20% or less? Were those lost to follow-up accounted for in the analysis?	NO / YES	NO / YES	NO / YES	NO / YES
10. Did the statistical methods examine changes in outcome measures from before to after the intervention? Were statistical tests done that provided p-values for the pre-to-post changes?	YES	YES	YES	YES
11. Were outcome measures of interest taken multiple times before the intervention and multiple times after the intervention?	NR	NR	NR	NR
12. If the intervention was conducted at a group level did the statistical analysis take into account the use of individual-level data to determine effects at the group level?	NA	NA	NA	NA
Total number of “YES”	8	6	7	6

CD, Cannot Determine; NA, Not Applicable; NR, Not Reported.

### Meta-analysis

**Fig. 2:**
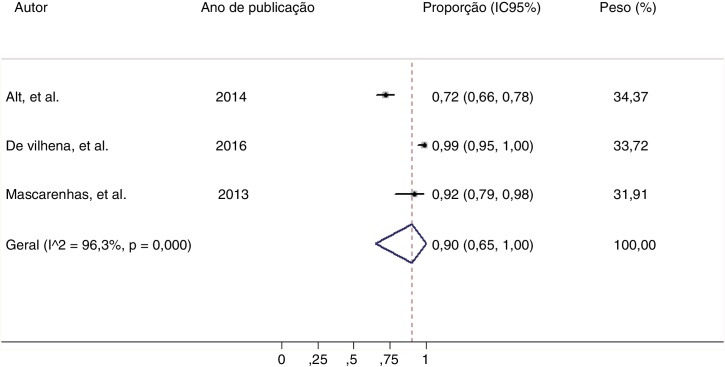
Meta-analysis of the proportion of patients with chronic rhinosinusitis who reported improved sleep quality after undergoing endoscopic nasal surgery. No overall data are available from one study.^20^ CI, Confidence Interval.

The percentage of patients with chronic rhinosinusitis who reported improved sleep quality after undergoing surgery was 90% (95% CI: 65%‒100%, I^2^ = 96.3%) (Fig. 2).^12^, ^20^, ^21^, ^22^ This is an overall measure of improvement. For each of the symptoms related to sleep quality, the improvement was lower (57%‒67%) (Table 3). However, these meta-analysis results showed high heterogeneity. Because of the low number of studies included, neither meta-regression nor publication bias analysis could be performed.

**Table 3:** Meta-analysis of the percentage of patients with chronic rhinosinusitis who reported an improvement in each of the five symptoms related to sleep quality after undergoing endoscopic sinus surgery.

Symptom description	% (95% CI)	*p*-value	I^2^ in %
Difficulty falling asleep	67 (56‒78)	< 0.01	84.1
Wake up at night	57 (19‒90)	< 0.01	98.6
Lack of a good night´s sleep	65 (50‒78)	< 0.01	90.4
Wake up tired	62 (40‒82)	< 0.01	95.6
Fatigue	60 (32‒84)	< 0.01	97.3

CI, Confidence Interval; I^2,^ Chi-Squared.

### Average scores before and after the surgery

Table 4^12^, ^20^, ^21^, ^22^ shows the mean scores for sleep symptoms before and after surgery. Improvement was observed in all items. In one of the studies, the summarized item scores decreased by 42.6%, from 13.5 preoperative to 7.7 postoperative.

**Table 4:** Percentage improvement in the mean scores on the SNOT-20 and SNOT-22 for sleep symptoms before and after surgery.

Author, year of publication	Item	Preoperative (SD)	Postoperative (SD)	Improvement (%)
Alt et al.^12^	Difficulty falling asleep	2.2 (1.6)	1.2 (1.4)	46.3
	Wake up at night	2.6 (1.6)	1.5 (1.4)	42.8
	Lack of a good night´s sleep	2.8 (1.6)	1.6 (1.5)	42.1
	Wake up tired	2.9 (1.5)	1.7 (1.5)	40.4
	Fatigue	2.9 (1.5)	1.6 (1.5)	42.7
	Summarized scores items	13.5 (6,9)	7.7 (6.6)	42.6^a^
	Difficulty falling asleep	2.5 (0.2)	0.9 (0.1)	64.5
	Wake up at night	2.6 (0.2)	1.3 (0.1)	51.7
De Vilhena et al.^21^	Lack of a good night´s sleep	2.6 (0.2)	1.1 (0.1)	59.5
	Wake up tired	2.7 (0.2)	1.0 (0.1)	64.0
	Fatigue	2.6 (0.2)	1.0 (0.1)	59.8
	Difficulty falling asleep	3.6 (1,8)	0.6 (1.1)	83.3
	Wake up at night	3.7 (1.6)	1.1 (1.4)	70.3
Mascarenhas et al.^22^	Lack of a good night´s sleep	3.6 (1.8)	0.8 (1.4)	77.8
	Wake up tired	2.9 (2.0)	0.6 (1.2)	79.3
	Fatigue	2.7 (1.9)	0.8 (1.3)	70.4
	Difficulty falling asleep	1.2 (1.0)	0.4 (0.6)	64.7
	Wake up at night	0.5 (0.6)	0.4 (0.6)	10.8
Li et al.^20^	Lack of a good night´s sleep	0.9 (0.9)	0.5 (0.5)	51.1
	Wake up tired	0.7 (1.0)	0.3 (0.5)	54.2
	Fatigue	0.6 (0.9)	0.4 (0.6)	39.7

SD, Standard deviation

a Only Alt et al.^12^ reported the summarized scores items.

## Discussion

The results of this study indicate that patients with chronic rhinosinusitis reported improved sleep quality after nasal endoscopic surgery. The overall sleep improvement (90% of the patients) (Fig. 2) was higher compared with the analysis of each individual symptom related to sleep quality separately (57% to 67% of the patients) (Table 3). This difference between the general and the specific is expected.

When evaluating a topic by a general criterion, the percentage tends to be higher than assessing each of its components in isolation, that is, each of the five questions related to sleep quality.

Alt et al. observed an improved sleep quality with surgical intervention, but not an improvement in apnea.^23^ Most studies available in the literature correlate nasal obstruction with sleep respiratory disorders but not with sleep quality.^24^, ^25^, ^26^ This may have been the reason for the limited number of studies identified in the literature search.

Of the four studies included in this systematic review, one study was conducted in North America (Canada and United States), one in South America (Brazil), one in Europe (Portugal) and one in Asia (China). The diversity of places where the studies were conducted expresses the cultural diversity of the subjects investigated, which may be one cause of the observed high heterogeneity. In addition, the characteristics of the samples between the studies were not homogeneous (Table 1). Whereas two studies^12^, ^20^ included recalcitrant cases, one study^21^ excluded patients with this type of rhinosinusitis. These differences between the studies may justify the high heterogeneity found in this meta-analysis.

The use of two questionnaires, with self-reported information of a subjective nature, may be a limiting factor for the study. These questionnaires aim to assess the quality of life of patients with chronic rhinosinusitis, not the quality of sleep. However, their use is justified by the fact that they present five items directly related to sleep quality and are the only ones available in the search results. In addition, the use of only the SNOT-20 and SNOT-22 instruments makes it possible to reduce the heterogeneity of the studies. This is only an assumption, as the small number of articles in the review prevents the exploration of possible sources of heterogeneity.

The results of this review should be interpreted with caution because they may be distorted by selection biases and confounding factors. The impact of poor sleep hygiene, medications and insomnia has been documented in the literature and should be considered as potential confounding factors to the study of the relationship between chronic rhinosinusitis and difficulty falling asleep.^27^ In addition, these results are based on a less robust design. No randomized clinical trial regarding chronic rhinosinusitis and sleep quality was found.

## Conclusion

In conclusion, this study revealed that nasal endoscopic surgery for patients with chronic rhinosinusitis seems to improve the quality of sleep and each of the symptoms related to it when using SNOT-20 or SNOT-22 to analyze pre- and post-operative. Because this conclusion is based on studies with high heterogeneity and use only SNOT-20 or SNOT-22 as an instrument of evaluation of sleep quality, more investigations may be justifiable to confirm the real impact of surgery on sleep quality.

## Conflicts of interest

The authors declare no conflicts of interest.
